# Chinese Medicine Syndrome Differentiation for Early Breast Cancer: A Multicenter Prospective Clinical Study

**DOI:** 10.3389/fonc.2022.914805

**Published:** 2022-07-07

**Authors:** Qianqian Guo, Meaghan E. Coyle, Anthony Lin Zhang, Xiaohong Xue, Weihe Bian, Aili Song, Xiaohong Xie, Ri Hong, Gang Lyu, Lifang Liu, Qianjun Chen, Charlie Changli Xue

**Affiliations:** ^1^ The Second Affiliated Hospital of Guangzhou University of Chinese Medicine, Guangdong Provincial Hospital of Chinese Medicine, Guangdong Provincial Academy of Chinese Medical Sciences, Guangzhou, China; ^2^ The China–Australia International Research Centre for Chinese Medicine, School of Health and Biomedical Sciences, Royal Melbourne Institute of Technology University, Melbourne, VIC, Australia; ^3^ Department of Mammary, Yueyang Hospital of Integrated Traditional Chinese and Western Medicine, Shanghai University of Traditional Chinese Medicine, Shanghai, China; ^4^ Department of Breast Diseases, The Affiliated Hospital of Nanjing University of Chinese Medicine, Nanjing, China; ^5^ Department of Breast Surgery, Affiliated Hospital of Shandong University of Traditional Chinese Medicine, Jinan, China; ^6^ Department of Breast Surgery, The First Affiliated Hospital of Zhejiang Chinese Medical University (Zhejiang Provincial Hospital of Traditional Chinese Medicine), Hangzhou, China; ^7^ Breast Department, Sanya Women and Children’s Hospital Managed by Shanghai Children’s Medical Centre, Sanya, China; ^8^ Department of Breast, Chongqing Hospital of Traditional Chinese Medicine, Chongqing, China; ^9^ Department of Breast Surgery, The First Hospital of Hunan University of Chinese Medicine, Changsha, China; ^10^ Breast Department, Guangdong Provincial Hospital of Chinese Medicine, The Second Affiliated Hospital of Guangzhou University of Chinese Medicine, Guangzhou, China

**Keywords:** Chinese medicine, syndrome differentiation, cluster analysis, early breast cancer, multicenter clinical study

## Abstract

**Background:**

Chinese medicine (CM) syndrome differentiation is one of the fundamental principles that guide the practice of Chinese herbal medicine (CHM). CHM has been widely used among breast cancer patients. Contemporary literature varies in syndrome diagnosis, and there is a need to standardize syndrome differentiation according to the different stages of breast cancer treatment. This multicenter clinical study aims to identify the CM syndromes and the clinical signs and symptoms in women with early breast cancer.

**Methods:**

Participants who met the inclusion and exclusion criteria were interviewed during the five treatment stages: preoperative, postoperative, chemotherapy, radiation therapy, and endocrine therapy. Patient demographic data and CM syndrome (as recorded by the treating CM clinicians in medical records) were gathered. Signs and symptoms were analyzed using descriptive statistics to derive the standardized CM syndromes using hierarchical cluster analysis.

**Results:**

The analysis included 964 interviews with 620 participants enrolled between April 29, 2020 and May 30, 2021 from eight participating hospitals in China. The two most frequent syndromes recorded in medical records were dual deficiency of *qi* and blood, and dual deficiency of *qi* and *yin* during all but the preoperative stage. The symptoms of lassitude, lack of strength, and insomnia were common in all but the preoperative stage. Cluster analysis identified two clusters in the preoperative stage that most closely resembled the syndrome diagnoses of liver stagnation with congealing phlegm, and dual deficiency of the liver and kidney. Two clusters—dual deficiency of *qi* and blood, and dual deficiency of *qi* and *yin*—were common to multiple treatment stages. The syndrome cluster of spleen and stomach disharmony existed in both the postoperative and chemotherapy stages. Cluster analysis of the radiation therapy stage identified the unique syndrome of *yin* deficiency with fire toxin, while the endocrine therapy included the syndromes of liver depression and kidney deficiency.

**Conclusions:**

This multicenter clinical study showed consistency between results from cluster analysis and the most common syndromes recorded in the medical records. Findings from this clinical study will be further validated in a Delphi study to standardize CM syndromes for various stages of breast cancer treatment.

**Clinical Trial Registration:**

www.chictr.org.cn/index.aspx, identifier ChiCTR2000032497.

## Introduction

Breast cancer is the most commonly diagnosed cancer among women (1). Early breast cancer is nonmetastatic, and the treatment goals include tumor eradication and recurrence prevention (2). Conventional medical treatment options are surgical intervention with or without radiation therapy, and systemic therapy, such as chemotherapy, targeted therapy, and endocrine therapy (2, 3). Many women seek additional treatment with complementary therapies, including Chinese medicine (CM) (4).

Chinese herbal medicine (CHM) has been used as an adjunctive therapy to improve the quality of life of breast cancer patients (4). The prescription of CHM formulas is based on syndrome differentiation, which is identified by synthesizing the patient’s signs and symptoms according to CM theory (5). Our systematic review (6) examined the syndrome distribution in the contemporary literature for early breast cancer and found variation in the existing evidence. Such variation leads to inconsistent use of Chinese herbal formula that necessitates standardizing the syndrome differentiation.

Previous clinical studies (7–10) have presented the CM syndromes observed in each of the different treatment stages. However, differences in the definitions of each treatment stage, and the single-center nature of the studies, have limited the generalizability of the findings. For example, some studies (7, 9, 10) defined the consolidation stage as the 5 years after the end of chemotherapy/radiation therapy, regardless of whether patients were receiving endocrine therapy. These studies did not specifically focus on endocrine therapy, which confounds the results.

Zhu (11) describes how research to standardize CM syndromes includes three elements: a literature review, a clinical epidemiological study, and a Delphi study of experts. Our systematic review of the literature (6) examined the syndrome distribution; now, a clinical study is needed to collect clinical information. This clinical study aims to address these gaps by involving multiple hospitals and investigating the CM syndromes and the signs and symptoms of breast cancer patients in five clearly defined treatment stages: preoperative, postoperative, chemotherapy, radiation therapy, and endocrine therapy.

## Materials and Methods

This non-interventional, prospective clinical study examined the CM syndromes, signs, and symptoms seen in patients with early breast cancer. To ensure the representativeness of the data, the clinical study was carried out in eight hospitals from different geographic regions in China: East China (Shandong, Jiangsu, Zhejiang, and Shanghai), South China (Guangdong and Hainan), Central China (Hunan), and Southwest China (Sichuan).

### Inclusion and Exclusion Criteria

The participants were screened according to the inclusion and exclusion criteria. The inclusion criteria were as follows: (1) histologically confirmed primary invasive breast cancer (Stage I–III, no distant metastases); (2) female over 18 years; (3) Eastern Cooperative Oncology Group (ECOG) performance status of 0–2 (12); and (4) able to understand the study and provide written informed consent. The ECOG performance status is an assessment of cancer patients’ ability to perform at work and in daily life. Scores range from zero (asymptomatic) to five (death) (13). A score of zero means fully active and able to carry on all pre-disease performance without restriction, while two means symptomatic but up and about for more than 50% of waking hours. The exclusion criteria were as follows: (1) serious accompanying diseases such as cardio-cerebrovascular or psychological disease; (2) receiving neoadjuvant therapy or receiving chemotherapy, radiation therapy, or endocrine treatment simultaneously; (3) complications of breast cancer treatment, such as postoperative wound infection, severe lymphoedema, and neutropenic fever or radiation pneumonia; and (4) pregnant or breastfeeding participants.

### Study Procedure

Patients were recruited from the inpatient or outpatient breast department of participating hospitals. As part of routine care, patients received conventional medical treatments after pathological confirmation of early breast cancer, including breast surgery, chemotherapy, radiation therapy, targeted therapy (for human epidermal growth factor receptor 2-positive patients), and endocrine therapy (3). The researcher checked the hospital medical records and surgery list for newly hospitalized patients to identify patients who met the inclusion criteria. For patients who attended the outpatient department, the medical records of the outpatient department were screened to identify the eligible participants. The details of the project were explained to ensure the participants fully understood before participating in this study.

Participants were informed that they would be interviewed up to a maximum of five times, once in each of the five treatment stages, depending on the treatment they were receiving at the time of recruitment. For example, women who were recruited in the preoperative stage could be interviewed five times (once in each of the five treatment stages), while women recruited while receiving chemotherapy were interviewed up to a maximum of three times (i.e., during chemotherapy, radiation therapy, and endocrine therapy stages). Women who agreed to participate in the research signed the informed consent form, and the first face-to-face interview was conducted (approximately 15–20 min duration). After the first interview, the next appointment time was arranged for women receiving other treatments (in the next stage of their treatment); women who had completed their treatment were not required to attend any further interviews. For instance, if the patients were first interviewed at the postoperative stage and did not need to receive chemotherapy, radiation therapy, and endocrine therapy, the patient would not be followed up. Many women were interviewed in only one treatment stage. Participants were enrolled sequentially, with each participant being allocated a unique participant number that would be used for all interviews and on all case report forms (CRFs).

An electronic copy of the research proposal, CRF, and informed consent form were provided to each participating hospital. Researchers at each hospital received training in the standard operating procedure, which included a detailed explanation of the study procedure, the process for obtaining informed consent and completing the CRF to ensure that researchers at each hospital understood the process correctly, and the standard interview technique. A unique identifier was used to distinguish which participants were recruited from each participating hospital. Participating hospitals were in regular contact with the coordinating hospital (Guangdong Provincial Hospital of Chinese Medicine) to resolve any issues promptly. Furthermore, a monthly meeting was conducted to summarize any issues arising from different hospitals, provide additional advice and update the number of participants, which ensured the accuracy and quality of the data and confirmed that the research process was being followed.

### Data Collection

The CRF included three parts: participant demographic information, breast cancer history and treatment (including the CM syndrome diagnosis and CHM formula use), and signs and symptoms. The demographic information (e.g., age, height, weight, and marital status) was completed by the researcher and was supplemented from patient medical records. Information about breast cancer history and treatment (e.g., surgery category, pathological stage, histology type, and ECOG status), syndrome diagnosis (as recorded by the treating clinician), and prescribed CHM formula were collected from patient medical records.

Pathological staging of breast cancer is determined based on multiple factors: the tumor size, lymph node involvement, and distant metastases (3). While some of these factors inform conventional medical treatment options, they are not used to inform CM syndrome diagnosis. Furthermore, pathological staging is performed at the time of surgery (14). Patients with early breast cancer are not restaged during their conventional medical treatment unless they exhibit signs and symptoms of metastasis. In such cases, patients would undergo additional clinical and diagnostic examination and may be diagnosed with metastatic breast cancer instead (if metastases are found). For example, if metastases were detected during the 5 years of endocrine therapy, patients were not enrolled in this study since their new diagnosis would mean they no longer meet the eligibility criteria. The pathological stage was collected for each participant.

A list of signs and symptoms was collated from our previous systematic review (6). Signs and symptoms related to the breast and local area included breast mass, pain, skin changes in the area surrounding the operation site, and limb involvement. Other CM traditional diagnosis categories related to the affected body part (e.g., skin, head and face, oropharyngeal, chest, abdomen, and limbs), sleep, mood, gynecology, fertility, urine and bowel movements, appearance of the tongue (e.g., color, tongue coat, and physical features), and palpation of the pulse (e.g., rate and strength).

The presence or absence of each sign and symptom was obtained directly from participants, while information about tongue and pulse diagnosis were collected from the participant’s medical record. The terminology for syndromes and features relating to the tongue and pulse was standardized according to the *Chinese Terms in Traditional Chinese Medicine and Pharmacy* (15), *Clinic Terminology of Traditional Chinese Medical Diagnosis and Treatment–Syndromes* (16), and *Differential Diagnosis of Traditional Chinese Medicine Symptoms* (17). The translation of the syndromes was based on the *Chinese Terms in Traditional Chinese Medicine and Pharmacy* (15), the *World Health Organization International Standard Terminologies on Traditional Medicine in the Western Pacific Region* (18), and the World Health Organization’s International Classification of Diseases 11 (19) (Supplementary Chapter Traditional Medicine Conditions) wherever possible, while for the signs and symptoms, the translations were only based on the first two sources (15, 18).

An electronic CRF was used for data collection in Guangdong Provincial Hospital of Chinese Medicine, while the other participating hospitals used a hard copy CRF. If the information was not available from medical records—for example, if the participant was not prescribed CHM—it was marked as missing and indicated as “not reported” in the CRF. At the coordinating hospital, data were entered directly into the electronic CRF by using EpiData software (EpiData Software, RRID: SCR_008485 version 3.1). Data from other hospitals were collected on hard copy CRF and entered into EpiData software when returned to the coordinating hospital for data entry, verification, and archival. Once the participating hospital commenced enrolment, the coordinating hospital checked the data within 1 month to ensure data accuracy and quality, which ensured that the coordinators at participating sites were trained to minimize the chance of errors in future participants. Data were double checked through double entry into the EpiData software. If inconsistencies were detected, the coordinating hospital checked the CRF with the participating hospital, and the original data would be corrected when necessary.

### Data Analysis

The following definitions, adapted from Guo and Chen (2015) (20), were used for the five conventional medicine treatment stages: preoperative stage (from surgical admission to the beginning of surgery), postoperative stage (from the end of surgery to the beginning of chemotherapy or other conventional medicine treatments), chemotherapy stage (from the beginning of chemotherapy to 2 weeks after the end of chemotherapy), radiation therapy stage (from the beginning of radiation therapy to 2 weeks after the end of radiation therapy), and endocrine therapy stage (from the beginning to the end of endocrine therapy).

SPSS software (IBM SPSS Statistics, RRID : SCR_016479 version 26.0) was used to conduct the data analysis. Patient demographic information, syndrome frequency, signs and symptoms, and CHM formula frequency were analyzed using descriptive statistics. Frequency analysis was conducted to determine the syndromes according to pathological staging for each conventional medical treatment stage. Shapiro–Wilk tests were performed to determine the normality distribution of the continuous variables. The mean and standard deviation were calculated for normally distributed continuous data, and the interquartile range (P_25_, P_50_, and P_75_) were calculated for data with an asymmetrical distribution (21). For qualitative variables, counts and proportions are presented.

Cluster analysis is a method to treat the sample as a class and select similar class members as one cluster. Cases with similar syndrome diagnoses were clustered into one group; therefore, the Q-type clustering that clustered the *sample* was applied for the clinical data (22). The number of clusters was decided based on three factors: the change in the agglomeration schedule coefficient, calculated using SPSS software; the information provided from the dendrogram (a two-dimensional diagram) (23–27); and the interpretability and meaningfulness for the clusters for clinical practice. The weight of each cluster’s signs and symptoms was calculated (28–30). The items were screened according to the weight. Finally, the syndrome name of each cluster was informed according to *Clinic Terminology of Traditional Chinese Medical Diagnosis and Treatment–Syndromes* (16) and determined after a thorough discussion with the research team.

### Sample Size Calculation

According to the principles of cross-sectional surveys (31), the formula for calculating the sample size is as follows: *n* = K × Q/P, where *n* = sample size, K = coefficient, P = estimated overall positive rate, and Q = 1 − P. Setting the *α* value at 0.05 and the tolerance at 0.2P (the tolerance is always between 0.1P and 0.2P), the K is 100. Our previous systematic review showed that the most frequent syndrome was dual deficiency of *qi* and blood; the sample size calculation was conducted based on the expected frequency of that syndrome. The probability of the most frequent syndrome (i.e., the estimated overall positive rate) is 63/449 (14%), where the number 63 represents the frequency of the most common syndrome and 449 represents the total number of syndromes. Based on this calculation, the estimated number of participants required was 614. The sample size required for each center was calculated based on each center’s early breast cancer data for 2019. Since the number of early breast cancer patients per annum varied among the participating centers, the number of participants recruited from each site also varied.

### Ethics Approval and Consent to Participate

This study was approved by the Ethics Committee of Guangdong Provincial Hospital of Chinese Medicine (approval no. ZE2020-048-01) and was registered with the Royal Melbourne Institute of Technology University Human Research Ethics Committee (registration no. 23259) before recruitment commenced. Other hospitals received ethics approval from their hospitals [Shanghai hospital approval no. 2021–031; Jiangsu hospital approval no. 2020NL–181–02; Shandong hospital approval no. (2020) Ethics Committee Review (036)–KY; Zhejiang hospital approval no. 2020–KL–145–01; Hainan hospital approval (number not stated in the approval document); Chongqing hospital approval no. 2020–KY–KS–LG; Hunan hospital approval no. HN–LL–ZFKY–2021–001–01]. All participants provided written informed consent.

## Results

**Figure 1:**
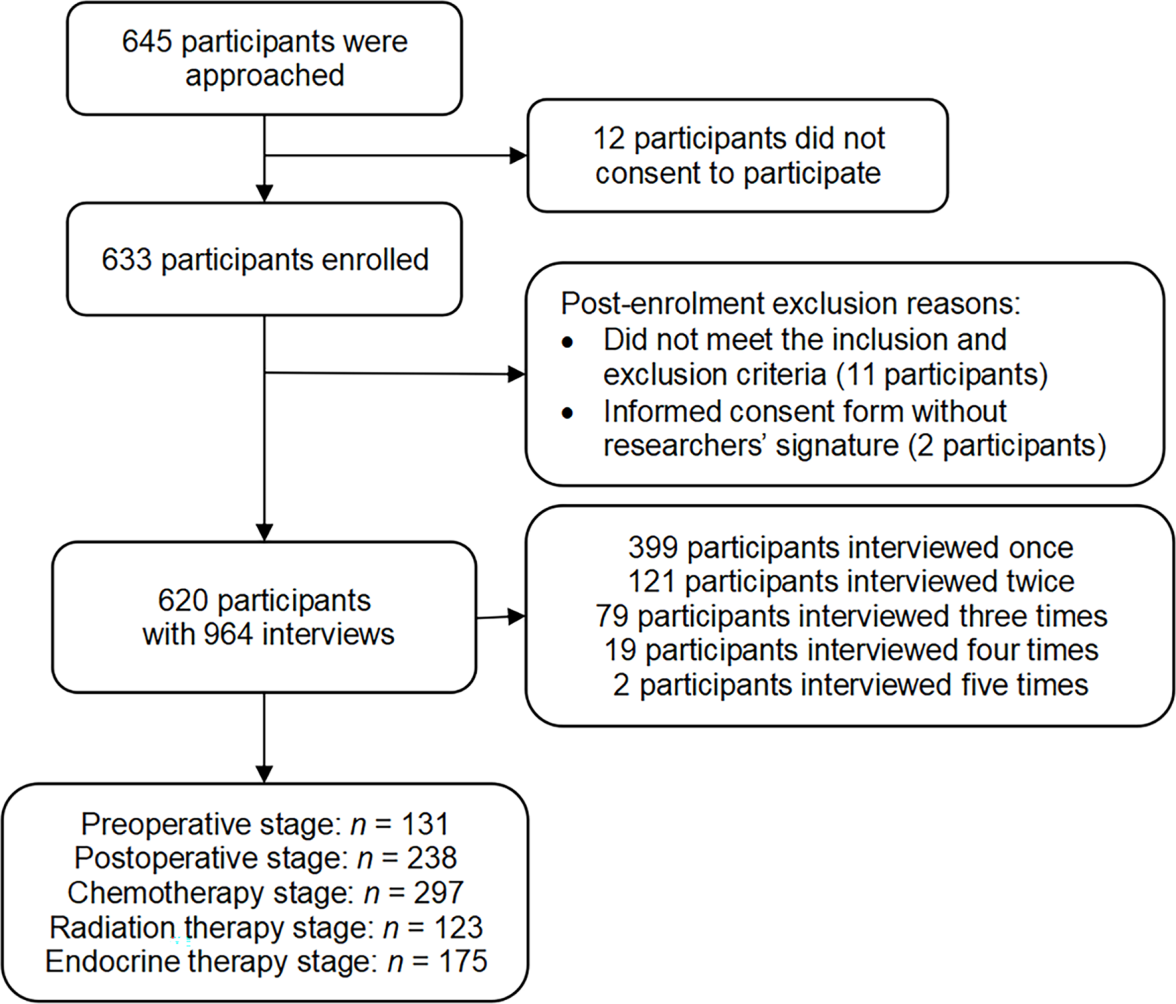
Flowchart of the participant inclusion. *n* represents the number of interviews.

In total, 645 participants were approached about participating in this study (see **Figure 1**). Six hundred and thirty-three participants were enrolled from April 29, 2020 to May 30, 2021, and the study was completed on May 30, 2021. As eligible participants were interviewed multiple times throughout their treatment, the number of interviews is larger than the number of participants. Some interviews were deemed ineligible after monitoring the CRFs because the participants did not meet the inclusion and exclusion criteria or the informed consent form was without the researchers’ signature (see **Figure 1**). Finally, 620 participants with 964 interviews were included in the analysis: 131 in the preoperative stage, 238 in the postoperative stage, 297 in the chemotherapy stage, 123 in the radiation therapy stage, and 175 in the endocrine therapy stage (see **Supplementary File 1** for the number of participants from each hospital).

### Patient Characteristics

Participants’ demographic information results are presented in **Table 1**. The median age of participants was 50 years. The median body mass index (BMI) was 22.9. Most enrolled participants were married (94.2%). Two hundred and sixty-three participants (42.4%) received conservation surgery, while 357 participants (57.6%) underwent a mastectomy. The majority of participants were diagnosed during pathological stage I (226 participants, 36.5%) or stage II (300 participants, 48.4%). Over 90% of participants were diagnosed with invasive ductal carcinoma.

**Table 1:** Participant characteristics.

Characteristic	Participants (*N* = 620)
**Age (years)**	
Median	50
Range	23–78
P_25_, P_75_	44, 58
**Height (cm)**	
Median	158
Range	140–176
Mean ± standard deviation	158.3 ± 5.8
**Weight (kg)**	
Median	58
Range	37–86
P_25_, P_75_	52, 63
**BMI (kg/m^2^)**	
Median	22.9
Range	15.2–36.3
P_25_, P_75_	20.8, 24.9
**Marriage status (*n*, %)**	
Single	22 (3.5)
Married	584 (94.2)
Divorced	5 (0.8)
Widowed	9 (1.5)
**Surgery category (*n*, %)**	
Conservation surgery	263 (42.4)
Mastectomy	357 (57.6)
**Pathological stage (*n*, %)**	
Stage I	226 (36.5)
Stage II	300 (48.4)
Stage III	94 (15.2)
**Histologic type (*n*, %)**	
Ductal	586 (94.5)
Lobular	7 (1.1)
Other	27 (4.4)
**ECOG performance status (*n*, %)**	
Score = 0	309 (49.8)
Score = 1	298 (48.1)
Score = 2	13 (2.1)

ECOG, Eastern Cooperative Oncology Group.

The age, height, weight, BMI, and marital status of participants in each treatment stage were similar to the overall sample (see **Supplementary File 2**). This provides some reassurance that the patients interviewed during each treatment stage were similar in terms of these demographic characteristics. For patient characteristics related to breast cancer and breast cancer treatment, some differences were noted in the type of surgery participants received. For example, while more women in the total sample underwent mastectomy, the rate of mastectomy was lower among women in the radiation therapy stage. The reason may be related to the fact that radiation therapy is a standard component of breast conserving surgery (32); for mastectomy, the application of radiation therapy should consider other factors, such as the positive lymph nodes (2).

Finally, one obvious difference was noted in participants’ ECOG performance status during the different treatment stages. During the preoperative stage, the vast majority (93.1%) of participants were assessed as having an ECOG score of 0, which means that patients are fully active and able to perform their daily activities to the same level as before the disease, without restriction. After surgery, only 23.9% of participants were assessed as having an ECOG score of 0; postoperatively, the majority of participants (75.2%) had an ECOG score of 1, which indicates that patients are mildly restricted in participating in strenuous activity, but can still perform light duties. During the chemotherapy stage, the number of participants with an ECOG score of 2 (ambulatory and capable of self-care but unable to work) increased to 1.7%, an increase from less than 1% preoperatively and postoperatively. This result is probably influenced by the known side effects of chemotherapy, including fatigue, nausea, and vomiting (33). It is likely that the ECOG performance status is related to CM syndrome diagnosis. However, since the syndromes in each conventional medicine treatment stage were not evenly distributed (see **Table 2**), we were unable to examine this relationship in a way that could provide meaningful results for clinical practice.

**Table 2:** The three most frequent Chinese medicine syndromes for each treatment stage.

Stage	Frequency (*n*)	Percentage (%)
**Preoperative**	(*n *= 131)	
Liver stagnation with congealing phlegm	104	79.4
Disharmony of *Chong* and *Ren* Vessels	19	14.5
Blood stasis with phlegm	3	2.3
**Postoperative**	(*n* = 238)	
Spleen and stomach disharmony	141	59.2
Dual deficiency of *qi* and blood	31	13.0
Dual deficiency of *qi* and *yin*	29	12.2
**Chemotherapy**	(*n* = 297)	
Dual deficiency of *qi* and blood	124	41.8
Spleen and stomach disharmony	63	21.2
Dual deficiency of *qi* and *yin*	42	14.1
**Radiation therapy**	(*n* = 123)	
Dual deficiency of *qi* and *yin*	39	31.7
Dual deficiency of *qi* and blood	35	28.5
*Yin* deficiency with fluid depletion	22	17.9
**Endocrine therapy**	(*n* = 175)	
Dual deficiency of *qi* and *yin*	33	18.9
Dual deficiency of the spleen and kidney	30	17.1
Dual deficiency of *qi* and blood	16	9.1

### Syndrome Frequency—From Clinical Records

The three most frequently reported syndromes, as recorded by clinicians in medical records, for each treatment stage are summarized in **Table 2**. The percentage in **Table 2** is calculated according to the number of interviews for each treatment stage. The frequency of all syndromes in each stage is available in **Supplementary File 3**. The frequency of syndromes for each pathological stage in each treatment stage is available in **Supplementary File 4**.

Eight syndromes were reported in the 131 interviews during the preoperative stage, of which liver stagnation with congealing phlegm (79.4%) was the most common syndrome. Spleen and stomach disharmony was the most frequent of the 10 syndromes identified from 238 interviews in the postoperative stage. Twenty-five syndromes were reported from 297 interviews in the chemotherapy stage, of which dual deficiency of *qi* and blood (41.8%) was the most common syndrome. For the radiation therapy stage, 123 interviews identified 13 syndromes, with dual deficiency of *qi* and *yin* (31.7%) reported most frequently. Syndromes were more diverse in the endocrine therapy stage, with 33 syndrome diagnoses from the 175 interviews. Dual deficiency of *qi* and *yin* (18.9%) was the most common syndrome.

Analysis of the syndromes by pathological stage showed that the number of participants with the most frequently diagnosed syndrome (Liver stagnation with congealing phlegm in the preoperative stage and Spleen and Stomach disharmony in the postoperative stage) was similar for those with pathological stage I and stage II breast cancer (see **Supplementary File 4**, **Tables S4.1 and S4.2**). This pattern differed in other treatment stages. In the chemotherapy stage, the number of women with the most common syndrome (dual deficiency of *qi* and blood) was higher in women with pathological stage II than stage I (23.6% versus 13.5%, respectively). In the radiation therapy stage, the number of women with the most common syndrome (dual deficiency of *qi* and *yin*) and with pathological stage II breast cancer was considerably higher than in women with pathological stage I breast cancer (17.1% versus 6.5%, respectively; see **Supplementary File 4**, **Table S4.4**). Finally, there was little difference in the number of women with the most common syndrome between pathological stage I and II (6.3% versus 10.3%, respectively) in the endocrine therapy stage.

### Chinese Herbal Medicine Formulas—From Medical Records

Among the 964 interviews from clinical medical records, there were 454 documented prescriptions of CHM formulas (see **Supplementary File 5**). In the preoperative stage, 66 prescriptions were reported from the 131 interviews. Only four CHM formulas were prescribed for the three syndromes that were documented in this stage. *Xiao yao lou bei san* was the most common formula, prescribed for liver stagnation with congealing phlegm. One hundred and seventy-four CHM prescriptions were reported from 238 interviews in the postoperative stage. The formula *Xiang sha liu jun zi tang* was the most common formula, prescribed 126 times for the syndrome spleen and stomach disharmony and 27 times for the syndrome spleen and stomach weakness.

There was more diversity in the CHM formulas prescribed and syndromes documented in the chemotherapy stage. In total, 103 CHM prescriptions were recorded from 297 interviews. The formula *Gui pi tang* was the most common formula for dual deficiency of *qi* and blood (prescribed 23 times), and it was sometimes used in combination with *Gui lu er xian tang* (prescribed 8 times). The radiation therapy stage included 49 CHM formula prescriptions from 123 interviews. Again, the formula *Gui pi tang*, prescribed for the syndrome dual deficiency of *qi* and blood (prescribed 28 times), was reported far more frequently than other formulas. The second most commonly used formula was *Shen mai san*, prescribed 10 times for the syndrome dual deficiency of *qi* and *yin*. As in the chemotherapy stage, there was more diversity in the CHM formulas prescribed in the endocrine therapy stage. Sixty-two CHM prescriptions were recorded from 175 interviews. The formulas in the endocrine therapy stage varied and most formulas were only prescribed only once. The formula *Huang qi jie du tang* was commonly used for deficiency of healthy *qi* and exuberance of pathogen (prescribed 10 times).

### Sign and Symptom Frequency—From Interviews

The 10 most frequently reported signs and symptoms for each treatment stage are summarized in **Table 3**. Signs and symptoms are presented as a frequency and percentage of the total number of participants included in each treatment stage. The most frequent symptoms in the preoperative stage—hard breast mass, the boundary of the mass is unclear, fixed breast mass, and the surface of the mass is not smooth—all relate to the clinical examination of the breast mass. Dysphoria, anger, stringy pulse, and vexation relate to liver stagnation. Lassitude, lack of strength, and insomnia were all present in the remaining four stages, except for the preoperative stage. Both lassitude and lack of strength were suggestive of *qi* deficiency, while insomnia—considered a deficient symptom in this instance—was related to heart, spleen, or kidney deficiency. Anorexia in the postoperative stage was indicative of spleen and stomach weakness or disharmony, while pain in the operation site was specific to the postoperative stage.

**Table 3:** Ten most frequently reported Chinese medicine signs and symptoms for each treatment stage.

Stage	Signs and symptoms	Frequency (*n*, %)
Preoperative	Stringy pulse	112 (85.5)
	Hard breast mass	95 (72.5)
	Dysphoria	85 (64.9)
	The boundary of the mass is unclear	82 (62.6)
	Anger	78 (59.5)
	Pale tongue	78 (59.5)
	Fixed breast mass	76 (58.0)
	White tongue coat	75 (57.3)
	Vexation	71 (54.2)
	The surface of the mass is not smooth	69 (52.7)
Postoperative	Thready pulse	182 (76.5)
	White tongue coat	163 (68.5)
	Dry mouth	144 (60.5)
	Lassitude	138 (58.0)
	Lack of strength	135 (56.7)
	Anorexia	128 (53.8)
	Pale tongue	128 (53.8)
	Thin tongue coat	124 (52.1)
	Insomnia	117 (49.2)
	Pain in operation area	111 (46.6)
Chemotherapy	Thready pulse	204 (68.7)
	Lack of strength	186 (62.6)
	White tongue coat	186 (62.6)
	Loss of hair	168 (56.6)
	Lassitude	166 (55.9)
	Dry mouth	143 (48.2)
	Thin tongue coat	136 (45.8)
	Insomnia	135 (45.5)
	Anorexia	131 (44.1)
	Pale tongue	129 (43.4)
Radiation therapy	Thready pulse	100 (81.3)
	Dry mouth	96 (78.1)
	Thin tongue coat	96 (78.1)
	Insomnia	76 (61.8)
	Lack of strength	75 (61.0)
	Lassitude	74 (60.2)
	Red tongue	70 (56.9)
	Dry throat	51 (41.5)
	Rapid pulse	51 (41.5)
	Pain of the radiated skin	40 (32.5)
Endocrine therapy	Thready pulse	116 (66.3)
	Thin tongue coat	94 (53.7)
	Dry mouth	84 (48.0)
	Lack of strength	84 (48.0)
	Lassitude	78 (44.6)
	White tongue coat	77 (44.0)
	Insomnia	74 (42.3)
	Tidal fever	73 (41.7)
	Deep pulse	59 (33.7)
	Pale tongue	57 (32.6)

In the chemotherapy stage, the most common signs and symptoms were loss of hair—suggestive of blood deficiency, kidney deficiency, or spleen deficiency—and anorexia, which was indicative of spleen and stomach deficiency. The main symptoms in the radiation therapy stage were dry mouth and dry throat, related to *yin* deficiency. Pain in the radiated skin was also a common symptom and was specific to this treatment stage. During the endocrine therapy stage, tidal fever was the most common symptom, which indicated *yin* deficiency. Tidal fever did not occur in the other treatment stages.

### Hierarchical Cluster Analysis

The cluster results for each stage of breast cancer treatment are summarized in **Table 4**. Dendrograms for each treatment stage are presented in **Supplementary File 6**.

**Table 4:** Hierarchical cluster analysis results by treatment stage.

Treatment stage	Syndrome	Signs and symptoms (weight)
Preoperative	Liver stagnation with congealing phlegm	The boundary of the mass is unclear (8), hard breast mass (8), fixed breast mass (8), the surface of the mass is not smooth (8), the skin color of the mass is unchanged (7), stringy pulse (7), dysphoria (6), anger (5), thin tongue coat (5), vexation (5), pale tongue (5), insomnia (4), dry mouth (4), white tongue coat (4), chloasma (3), depression (3), bitter taste in mouth (2), slippery pulse (2), menstrual blood clot (2), soreness of loins (2), profuse sweating (2).
	Dual deficiency of the liver and kidney	Stringy pulse (10), white tongue coat (8), pale tongue (7), anger (7), dysphoria (6), insomnia (6), vexation (6), hard breast mass (6), dry mouth (5), thin tongue coat (5), chloasma (5), soreness of loins (4), depression (4), profuse sweating (4), thready pulse (3), the boundary of the mass is unclear (3), pink tongue (3), speechlessness (3), greasy tongue coat (3), bitter taste in mouth (3).
Postoperative	Dual deficiency of *qi* and *yin*	Thready pulse (10), pink tongue (10), white tongue coat (10), thin tongue coat (9), lassitude (7), lack of strength (7), dry mouth (7), pain in the operation area (5), insomnia (5), soreness of loins (5), dry throat (4), anorexia (4), contralateral breast nodules or lumps (4), stabbing pain in the operation area (4), pain in the throat (3), cough (3), loss of taste (3).
	Spleen and stomach disharmony	Pale tongue (9), white tongue coat (9), anorexia (9), slippery pulse (8), thready pulse (7), dry mouth (7), profuse sweating (6), pain in the operation area (6), insomnia (5), difficulty in defecation (5), constipation (4), thin tongue coat (4), greasy tongue coat (4), teeth-marked tongue (4), soreness of loins (3), pink tongue (3), pain in the throat (3), chloasma (3), dry throat (2), sallow complexion (2).
	Dual deficiency of *qi* and blood	Lack of strength (9), lassitude (9), thready pulse (8), pale tongue (7), dry mouth (6), insomnia (5), anorexia (5), white tongue coat (5), thin tongue coat (5), profuse sweating (5), soreness of loins (4), pain in the operation area (4), dizziness (4), constipation (3), difficulty in defecation (3), slippery pulse (3), chloasma (3), teeth-marked tongue (3), spontaneous sweating (2), dry throat (2), pain in the throat (2), greasy tongue coat (2), red tongue (2).
Chemotherapy	Dual deficiency of *qi* and blood	White tongue coat (15), thready pulse (9), loss of hair (8), dry mouth (8), lack of strength (7), pink tongue (7), forgetfulness (7), numbness of the operation area (6), pale tongue (6), anorexia (6), thin tongue coat (5), numbness of the upper limb of the affected side (5), insomnia (5), sallow complexion (5).
	Kidney *yin* deficiency	Thready pulse (10), lack of strength (10), lassitude (10), thin tongue coat (8), insomnia (7), loss of hair (6), dry mouth (6), pale tongue (6), dizziness (5), profuse sweating (4), shortage of *qi* (4), white tongue coat (4), red tongue (3), chloasma (3), dark tongue (3), deep pulse (3), soreness of loins (3), pale complexion (2), tidal fever (2).
	Spleen and stomach disharmony	Anorexia (9), white tongue coat (8), loss of hair (8), thready pulse (7), lassitude (7), nausea (7), lack of strength (7), retch (5), dry mouth (5), insomnia (5), pale tongue (5), slippery pulse (4), thin tongue coat (4), pink tongue (4), loose stool (3), teeth-marked tongue (3), dizziness (3), loss of taste (3), bitter taste in mouth (2).
Radiation therapy	Dual deficiency of *qi* and blood	Thready pulse (12), pale tongue (12), lack of strength (11), lassitude (10), thin tongue coat (8), white tongue coat (8), dry mouth (7), insomnia (7), deep pulse (5), pale complexion (4), profuse sweating (4), forgetfulness (4), dizziness (4), pale-colored eyelid (3), teeth-marked tongue (3).
	Dual deficiency of *qi* and *yin*	Red tongue (11), lack of strength (11), lassitude (11), dry mouth (11), thin tongue coat (11), thready pulse (10), insomnia (8), dry throat (7), rapid pulse (6), profuse sweating (5), dryness of the radiated skin (3), tidal fever (3), forgetfulness (2).
	*Yin* deficiency with fire toxin	Dry mouth (10), thin tongue coat (9), red tongue (9), pain of the radiated skin (9), thready pulse (8), insomnia (7), rapid pulse (7), dry throat (7), redness of radiated skin (6), constipation (5), itching of the radiated skin (5), pain in the throat (4), dryness of the radiated skin (3), vexation (3), dry tongue (3), bitter taste in the mouth (3), profuse sweating (2).
Endocrine therapy	Dual deficiency of the spleen and kidney	Thready pulse (11), lassitude (10), lack of strength (10), thin tongue coat (9), dry mouth (6), deep pulse (6), insomnia (5), pale tongue (5), white tongue coat (4), soreness of loins (4), tidal fever (4), forgetfulness (4), anorexia (3), dark tongue (3), sallow complexion (3), profuse sweating (3), red tongue (3), shortage of *qi* (3), dizziness (3).
	Liver depression and kidney deficiency	Pale tongue (8), dysphoria (7), vexation (6), lack of strength (6), tidal fever (6), white tongue coat (5), stringy pulse (5), numbness of the limbs (5), anger (5), insomnia (5), profuse sweating (5), teeth-marked tongue (5), forgetfulness (4), slippery pulse (4), thready pulse (4), greasy tongue coat (4), lassitude (4), dry mouth (4), arthralgia (4), thin tongue coat (3), depression (3).
	Dual deficiency of *qi* and *yin*	Thready pulse (10), pink tongue (8), dry mouth (8), thin tongue coat (8), white tongue coat (8), tidal fever (7), amenorrhea (7), insomnia (7), arthralgia (6), sallow complexion (6), forgetfulness (5), deep pulse (5), numbness of the upper limb of the affected side (5), profuse sweating (4), moistened tongue (4).

#### Preoperative Stage

Two syndromes were summarized in the preoperative stage: liver stagnation with congealing phlegm, and dual deficiency of the liver and kidney. The signs and symptoms in these two clusters are quite similar; therefore, the different symptoms and the weight of the symptoms bring more guidance to distinguish the clusters as distinct syndromes. The first cluster—liver stagnation with congealing phlegm—has the highest weighted symptoms related to the breast mass and is followed by stringy pulse, dysphoria, anger, and vexation. The second cluster—dual deficiency of the liver and kidney—included other symptoms, such as thready pulse and speechlessness, which indicate deficiency syndrome. Furthermore, the soreness of loins was listed with a higher weight compared to the first cluster. These results suggest that the second syndrome is dual deficiency of the liver and kidney.

#### Postoperative Stage

The signs and symptoms in each cluster of the postoperative stage are internally consistent. The first cluster—dual deficiency of *qi* and *yin*—illustrated several closely related signs and symptoms, such as dry mouth, dry throat, pain in the throat, and cough, which indicate deficiency of *yin*, while lassitude and lack of strength referred to deficiency of *qi*. The second cluster—spleen and stomach disharmony—was associated with specific symptoms, for example, anorexia, slippery pulse, difficulty in defecation, constipation, greasy tongue coat, teeth-marked tongue, and sallow complexion. The symptom anorexia had equal highest weighting in this cluster. Even though the symptoms pain in the throat and dry throat were shown in this cluster, these symptoms had comparatively lower weighting and less influence on the cluster syndrome name. The third cluster—dual deficiency of *qi* and blood—showed that the symptoms lack of strength and lassitude had equal highest weighting; in addition, dizziness and spontaneous sweating indicated dual deficiency of *qi* and blood. The symptom soreness of loins was common to all three clusters. In the postoperative treatment stage, soreness of the loins is less likely to indicate kidney deficiency, and more likely to be the consequence of the requirement to lie down for a prolonged time after surgery.

#### Chemotherapy Stage

The signs and symptoms of the first cluster highly echoed those in the syndrome dual deficiency of *qi* and blood. The second cluster was named kidney *yin* deficiency syndrome because it included several representative symptoms (e.g., loss of hair, dizziness, soreness of loins, tidal fever, red tongue, thin tongue coat, and thready pulse) (16). Some symptoms, such as shortage of *qi* and pale complexion, seemed more indicative of *qi* and blood deficiency; however, when considering all the signs and symptoms, kidney *yin* deficiency was considered the most appropriate syndrome for this cluster. The third cluster, including symptoms such as anorexia, nausea, retch, and loose stool, suggested the syndrome spleen and stomach disharmony. Some symptoms—such as dizziness and bitter taste in the mouth—appeared with lower weighting, indicating that these were not the main symptoms, and their inclusion in the cluster did not change the decision about the syndrome name.

#### Radiation Therapy Stage

The first cluster—with the symptoms lack of strength, lassitude, pale complexion, dizziness, and pale-colored eyelid—indicated dual deficiency of *qi* and blood syndrome (16). The teeth-marked tongue—more commonly related to spleen deficiency—appears contradictory to this syndrome, but it had a lower weighting that did not influence the syndrome name. The second cluster, dual deficiency of *qi* and *yin*, included symptoms such as lassitude, lack of strength, dry mouth, and dry throat that were highly internally consistent (16). The symptom dryness of the radiated skin is specific to this treatment stage. The third cluster showed more symptoms of radiated skin: pain of the radiated skin, redness of the radiated skin, itching of the radiated skin, and dryness of the radiated skin. Compared to the second cluster, the third cluster was easier to distinguish, with more severe signs and symptoms related to *yin* deficiency and fluid consumption, such as constipation, dry tongue, and bitter taste in the mouth.

#### Endocrine Therapy Stage

The first cluster with the highest weighted symptoms is related to a deficiency syndrome. Symptoms such as anorexia, sallow complexion, soreness of loins, and dark tongue had lower weightings, and this cluster suggests dual deficiency of the spleen and kidney when all the signs and symptoms are considered. The second cluster—with symptoms of dysphoria, vexation, numbness of the limbs, anger, forgetfulness, and depression—indicated liver depression and kidney deficiency. The symptom depression had the lowest weighting, but it remains relevant to liver depression. The third cluster showed a higher weight for some symptoms, such as dry mouth and tidal fever, and most of the signs and symptoms indicated dual deficiency of *qi* and *yin*.

## Discussion

This clinical study is the first multicenter study published in English that identifies the CM syndromes seen in clinical practice for each of the stages of early breast cancer treatment. The dual deficiency of *qi* and blood syndrome and the dual deficiency of *qi* and *yin* syndrome were commonly seen in multiple treatment stages from both clinical medical records and cluster analysis results. Similarities and differences in the most common syndromes were observed between those from the medical records and outcomes from the hierarchical cluster analysis. The symptoms lassitude, lack of strength, and insomnia were commonly present in all but the preoperative stage.

While demographic information, such as age (34), weight, and BMI, may affect syndrome differentiation, these are not major factors determining a clinical CM syndrome diagnosis. Syndrome diagnosis is based on the presenting signs and symptoms (35), some of which may be related to age, weight, or BMI; however, such characteristics are not the primary consideration for syndrome diagnosis. Furthermore, while age and weight may be considered when planning conventional medical treatment for breast cancer, these factors are less important for planning Chinese medicine treatments.

The frequencies of clinician-recorded syndromes according to pathological stage were skewed toward the most frequently recorded syndromes. This information provides insights for CM clinicians for the types of syndromes they may expect to see for each pathological stage and during each medical treatment stage. However, these results do not prove causality, as this study was not designed to assess such associations. Additional research is needed to determine the associations between pathological stage and CM syndrome diagnoses and examine the clinical relevance of such findings.

Differences were detected between the syndromes recorded in medical records and those identified through cluster analysis results. Despite having different syndrome names, the groups of signs and symptoms share similar characteristics. For example, *yin* deficiency with fluid depletion from the medical records and *yin* deficiency with fire toxin from the cluster analysis in the radiation therapy stage both included the concept of *yin* deficiency. Compared to our systematic review—which reviewed journal articles, clinical textbooks, and clinical care documents (the documents that CM hospitals in China developed to outline disease treatment and improve clinical management)—the most common syndromes identified in this clinical study are somewhat similar to the syndromes in clinical textbooks and journal articles but more similar to the results from the clinical care documents (6). Logically, the clinical care documents are generated based on the published literature (journal articles and clinical textbooks), but take clinical practice and clinical experiences into consideration. These documents will influence and guide clinical practice, which explains why the clinical syndrome diagnoses are more likely to match those listed in the clinical care documents. For example, the three most common syndromes in the preoperative stage from clinical medical records (documented by the treating CM clinicians) were liver stagnation with congealing phlegm, disharmony of *Chong* and *Ren* vessels, and blood stasis with phlegm; these three syndromes were also the most frequently cited syndromes in clinical care documents.

Analysis of the CHM formula prescriptions showed that some Chinese herbal formulas were used for multiple different syndromes. For example, the formula *Ba zhen tang*—which is traditionally indicated for the dual deficiency of *qi* and blood—was used for *qi* stagnation and blood stasis in the postoperative stage and deficiency of healthy *qi* and exuberance of toxin in the endocrine stage. The formula *Shen ling bai zhu san* was applied for the dual deficiency of *qi* and blood in the postoperative and endocrine therapy stages and for dual deficiency of *qi* and *yin* in the radiation therapy stage; this formula is traditionally used for spleen deficiency with dampness encumbrance. The formula *Si jun zi tang*, which is usually prescribed for the syndrome spleen and stomach *qi* deficiency, was also used for various syndromes: dual deficiency of *qi* and blood in the postoperative, chemotherapy, and radiation therapy stage; and deficiency of healthy *qi* and exuberance of toxin, liver depression and spleen deficiency, spleen and kidney deficiency, and spleen *qi* deficiency in the endocrine therapy stage. Although most of the formulas used for syndromes other than those for which they are traditionally indicated were used with low frequency, this result revealed the disagreement in clinical practice and suggests the need to further standardized research on CHM formulas.

Unsurprisingly, the most common signs and symptoms of each stage were characteristic of the main syndromes recorded in medical records and obtained from cluster results in that stage. For instance, the signs and symptoms in the preoperative stage mostly suggested liver depression. In addition to the symptoms of lassitude and lack of strength related to cancer fatigue, insomnia was also frequently reported. Previous literature showed that nearly 80% of women undergoing chemotherapy for breast cancer experience insomnia (36). This symptom is also common during endocrine therapy (37), and was common in women in this study. The most common symptoms during radiation therapy included the specific symptoms that usually occur as side effects of radiation therapy (i.e., pain of the radiated skin). The remaining signs and symptoms—such as dry mouth, dry throat, red tongue, and thready and rapid pulse—indicate dual deficiency of *qi* and *yin*, which was the most common syndrome from medical records and one of the syndromes from cluster analysis. For the endocrine therapy stage, the symptom that appears to be specific to this stage is tidal fever, which is a common side effect of endocrine therapy treatments (38). A previous phase III trial reported that grade 3 hot flashes occurred in 4.7% of breast cancer patients receiving tamoxifen; this side effect was more common when tamoxifen was combined with ovarian function suppression (16.1%) (39).

The statistical methods for studying CM syndromes include regression analysis, principal component analysis, factor analysis, association analysis, decision tree, cluster analysis, and artificial neural network (40, 41). Huang et al. (42) conducted a retrospective study of 2,738 breast cancer cases from the China Medical University Hospital database. Neural network methods and cluster analysis identified liver–gallbladder dampness–heat as the primary syndrome. However, this study is a retrospective study, which may have inherent limitations for data collection, such as accuracy recording and comprehensiveness of symptom assessment. Furthermore, the study did not focus on the different stages of breast cancer. The results of Huang et al.’s research differ from our clinical study results, possibly because of different inclusion criteria, research methods, or statistical methods. Several cluster analysis studies have calculated the weight of individual signs and symptoms in several CM syndrome research (28–30); this approach was also used in this clinical study. The higher the weight, the more critical the signs and symptoms are for that syndrome. Multivariate statistical methods require multiple choices for data analysis in syndrome research, and different methods may produce different results.

Our previous systematic review (6) examined the literature from a variety of sources, including published research, clinical textbooks, and hospital clinical care documents, that were published before November 2019. Given that not all of these sources listed the syndromes, signs, and symptoms based on prospectively collected clinical data, we considered it critical to ensure that current clinical practice matches with the published information. We were pleased to find that, in most cases, there was significant overlap. This finding is important to reassure clinicians that the available literature seems to be as relevant today as it was when it was published. Furthermore, prospective data collection in this study overcomes several limitations of retrospective reports, including the accuracy of data recording and the comprehensiveness of symptom assessment.

This prospective study is an essential part of research to standardize CM syndromes and plays an important role in complementing and confirming our understanding of the syndromes, signs and symptoms, and CHMs used in the various stages of conventional medical treatment of early breast cancer. This study gathered real-world data through face-to-face interviews with breast cancer patients and is a critical step to determine the signs, symptoms, and syndromes that exist in real patients. The findings from this study will be integrated with those from the systematic review (6) for expert clinical judgment in the subsequent Delphi study.

This clinical study examined the clinical practice with a scientifically rigorous design and reliable data, which will provide a more accurate picture of real-world clinical presentations. Additionally, this study involved eight hospitals located in various regions of China. Participating centers varied in size and, therefore, in the number of early breast cancer patients seen per year. The maximum recruitment target was calculated based on the number of early breast cancer patients attending each participating center in 2019. The advantage of conducting a multicenter study is the larger number of participants that can be recruited (43). Generally, participating hospitals in multicenter studies come from different geographic locations, allowing for a diverse population group that ensures that the data are more representative (43).

Higher representativeness of the data facilitates the translation of research findings into clinical practice. In this study, the unequal distribution in the number of participants and interviews among the participating hospitals may undermine representativeness. Several Chinese studies have suggested that the geographical location influenced the CM syndrome diagnoses (44, 45); however, there is little literature on this topic that has been published in English. In CM theory, treatment may be tailored according to the local conditions, which suggests that the geographical location could influence the choice of CHM. Whether the geographical location could influence syndrome diagnosis needs more research.

The number of interviews varied among the different treatment stages, reflecting the diverse treatment courses taken by women with early breast cancer. Furthermore, the number of interviews in the preoperative and radiation therapy stages was relatively small compared to the other three stages. In participating hospitals, patients who are undergoing radiation therapy may be admitted to the radiation department rather than inpatient breast department. This may have resulted in some potentially eligible patients not being identified or enrolled in the study, which may reduce the number of participants in that stage and influence the results. The subgroup analysis of this clinical study, such as according to different participating hospitals, was not conducted for two reasons. First, the data for each treatment stage from the different hospitals was not equally distributed, which could influence the data representativeness in subgroup analysis. Second, subgroup analysis to compare the data from different hospitals was not the primary aim of this study since it is not so critical for CM syndrome standardized research. Multiple statistical methods could be applied in CM syndrome research to obtain different results, and identifying the most suitable method based on the available data is challenging. The application of the cluster analysis in this study is based on the research question and aims. One limitation is that this method cannot distinguish the primary and secondary signs and symptoms for each syndrome, which may limit the interpretation of the results.

The patients’ reporting of whether the signs and symptoms were present or absent was subjective. In this study, patients had no motivation to respond inaccurately; however, it is possible that they did, and this a limitation of the study. To ensure that the conduct of the interviews was standardized across all participating sites, the researcher (QG) conducted regular quality assurance training to ensure standardized data collection. Finally, given that CM syndrome diagnoses are fluid, not static like a conventional medical diagnosis of breast cancer, assessing the change in syndromes over time (for women who were interviewed multiple times) does not predict future syndromes. We attempted to conduct such analyses for women who participated in multiple interviews in the different treatment stages, but mapping syndrome trajectories is difficult because the progression of CM syndromes is not linear. In any case, the CM syndrome diagnosis is made for each individual according to their presenting signs and symptoms, not based on predictions about future syndromes. This personalized approach is one of the strengths of CM.

Our research is based on the different conventional treatment stages, which is a novel aspect of our research in integrating conventional medical treatment into our analysis of syndrome differentiation. Furthermore, we will focus on the integrative Chinese and Western research to examine the syndromes with different molecular subtypes of breast cancer.

## Conclusion

The results of this clinical study reflect the real-world clinical presentations of women with early breast cancer. Some similarities and differences were identified between the syndromes documented in medical records and those identified through cluster analysis. Dual deficiency of *qi* and blood, and dual deficiency of *qi* and *yin* were the most common syndromes from medical records and in cluster analysis among the various stages. The most frequent signs and symptoms in each stage were generally characteristic of the syndromes in that stage. The findings from this study will inform further study to develop consistent clinical decision-making for CM care of patients with early breast cancer.

## Data Availability Statement

The original contributions presented in the study are included in the article/**Supplementary Material**. Further inquiries can be directed to the corresponding authors.

## Ethics Statement

The studies involving human participants were reviewed and approved by Ethics Committee of Guangdong Provincial Hospital of Chinese Medicine. The patients/participants provided their written informed consent to participate in this study.

## Author Contributions

QG developed the study protocol, conducted the clinical study, coordinated with the participating hospitals, conducted interviews with women at the coordinating hospital, acquired and analyzed the data, and wrote and edited the manuscript. MC designed the study, analyzed and interpreted the results, provided comments during the process, and critically edited the manuscript. AZ provided comments on the study design and during the trial, and contributed to data interpretation and manuscript revision. QC and CX provided valuable comments for the study design and gave constructive opinions about the study results and the manuscript. QC also engaged with the multiple participating hospitals. XXu, WB, AS, XXi, RH, GL, and LL were the principal researchers in each hospital. These authors obtained ethical approval in each hospital, contributed to participant recruitment, provided feedback about the study procedure, and commented on the manuscript. All authors are accountable for all aspects of the work and approve the final version of the manuscript accepted for publication.

## Funding

This study has been supported by the Traditional Chinese Medicine Bureau of Guangdong Province (Research Project no. 20212079), the China–Australia International Research Centre for Chinese Medicine, and PhD scholarship support to the first author provided by the School of Health and Biomedical Sciences, Royal Melbourne Institute of Technology University.

## Conflict of Interest

The authors declare that the research was conducted in the absence of any commercial or financial relationships that could be construed as a potential conflict of interest.

## Publisher’s Note

All claims expressed in this article are solely those of the authors and do not necessarily represent those of their affiliated organizations, or those of the publisher, the editors and the reviewers. Any product that may be evaluated in this article, or claim that may be made by its manufacturer, is not guaranteed or endorsed by the publisher.

## Abbreviations

BMI, body mass index; CHM, Chinese herbal medicine; CM, Chinese medicine; CRF, case report form; ECOG, Eastern Cooperative Oncology Group.
