# Supplementary figures and images for: Chinese Medicine Syndrome Differentiation for Early Breast Cancer: A Multicenter Prospective Clinical Study
Source: Front Oncol. 2022 Jul 7;12:914805. doi: 10.3389/fonc.2022.914805 (PMC9300931; doi:10.3389/fonc.2022.914805)

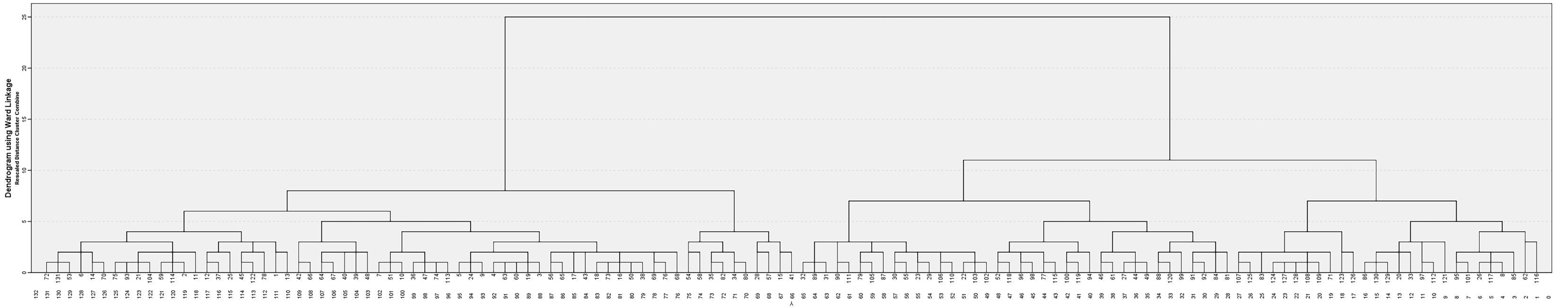

Supplement: Supplementary file 1 [file DataSheet_1.zip › Supplementary File 6_300 dpi/Preoperative.tif]

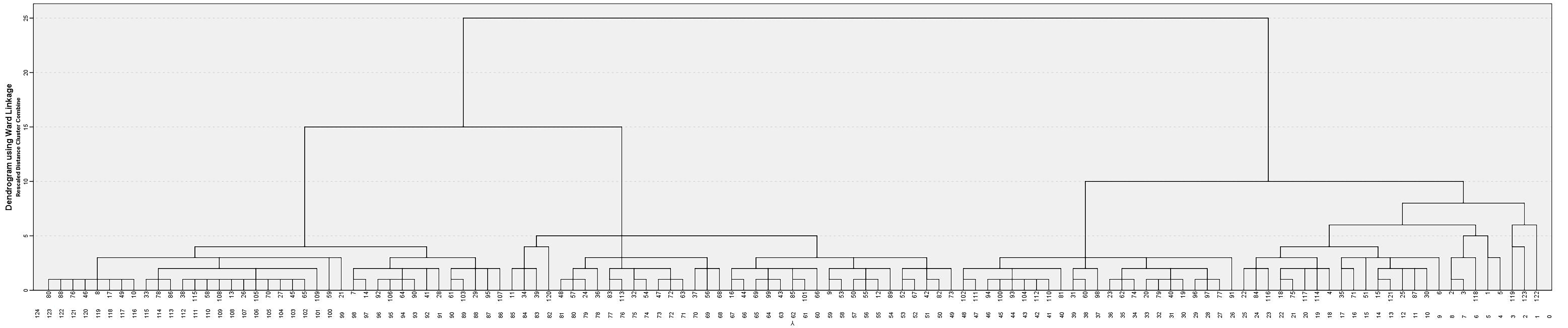

Supplement: Supplementary file 1 [file DataSheet_1.zip › Supplementary File 6_300 dpi/Radiation therapy.tif]
