# Supplementary material for: Chinese Medicine Syndrome Differentiation for Early Breast Cancer: A Multicenter Prospective Clinical Study
Source: Front Oncol. 2022 Jul 7;12:914805. doi: 10.3389/fonc.2022.914805 (PMC9300931; doi:10.3389/fonc.2022.914805)
Supplement: Supplementary file 2 [file Table_1.docx]

Supplementary File 1: Number of participants from each participating hospital.

Table S1.1. Number of participants from each hospital in each treatment stage.

| **Hospital** | **Number of participants (in total)** | **Number of participants (valid)** | **Number of interviews (valid)** | **Number of interviews in each treatment stages** | | | | |
| --- | --- | --- | --- | --- | --- | --- | --- | --- |
|  |  |  |  | **Preoperative** | **Postoperative** | **Chemotherapy** | **Radiation therapy** | **Endocrine therapy** |
| Guangdong Provincial Hospital of Chinese Medicine (Coordinating hospital) | 409 | 409 | 739 | 122 | 219 | 191 | 117 | 90 |
| Yueyang Hospital of Integrated Traditional Chinese and Western Medicine, Shanghai University of Traditional Chinese Medicine | 53 | 53 | 53 | 0 | 0 | 36 | 0 | 17 |
| The Affiliated Hospital of Nanjing University of Chinese Medicine | 50 | 50 | 50 | 0 | 1 | 12 | 2 | 35 |
| Affiliated Hospital of Shandong University of Traditional Chinese Medicine | 47 | 40 | 46 | 4 | 6 | 32 | 1 | 3 |
| The First Affiliated Hospital of Zhejiang Chinese Medical University (Zhejiang Provincial Hospital of Traditional Chinese Medicine) | 25 | 22 | 22 | 0 | 1 | 12 | 0 | 9 |
| Sanya Women and Children’s Hospital | 22 | 22 | 23 | 0 | 2 | 10 | 2 | 9 |
| Chongqing Hospital of Traditional Chinese Medicine | 12 | 12 | 19 | 5 | 9 | 4 | 0 | 1 |
| The First Hospital of Hunan University of Chinese Medicine | 15 | 12 | 12 | 0 | 0 | 0 | 1 | 11 |
| In total | 633 | 620 | 964 | 131 | 238 | 297 | 123 | 175 |
