# Supplementary material for: Chinese Medicine Syndrome Differentiation for Early Breast Cancer: A Multicenter Prospective Clinical Study
Source: Front Oncol. 2022 Jul 7;12:914805. doi: 10.3389/fonc.2022.914805 (PMC9300931; doi:10.3389/fonc.2022.914805)
Supplement: Supplementary file 3 [file Table_2.docx]

Supplementary File 2: Participant characteristics in each breast cancer treatment stage.

Table S2.1. Participant characteristics.

| **Characteristic** | **Preoperative (*n* = 131)** | **Postoperative (*n* = 238)** | **Chemotherapy (*n* = 297)** | **Radiation therapy (*n* = 123)** | **Endocrine therapy (*n* = 175)** | **Total sample**  **(*N* = 620)** |
| --- | --- | --- | --- | --- | --- | --- |
| Age (years) |  |  |  |  |  |  |
| Median | 50 | 50 | 50 | 49 | 50 | 50 |
| Range | 25–75 | 25–75 | 23–77 | 26–71 | 25–78 | 23–78 |
| P25, P75 | 42, 59 | 44, 58 | 44, 58 | 43, 57 | 43, 57 | 44, 58 |
| Height (cm) |  |  |  |  |  |  |
| Median | 157 | 157 | 158 | 157 | 159 | 158 |
| Range | 144–171 | 140–174 | 143–175 | 143–171 | 142–176 | 140–176 |
| Mean ± SD | 157.0 ± 5.8 | 157.3 ± 5.6 | 158.0 ± 5.9 | 157.1 ± 5.7 | 159.0 ± 6.0 | 158.3 ± 5.8 |
| Weight (kg) |  |  |  |  |  |  |
| Median | 57 | 56 | 57 | 58 | 58 | 58 |
| Range | 40–86 | 40–86 | 38–83 | 41–82 | 37–80 | 37–86 |
| P25, P75 | 50, 62 | 51, 63 | 50.5, 63 | 53, 63 | 52, 63 | 52, 63 |
| BMI (kg/m2) |  |  |  |  |  |  |
| Median | 22.9 | 22.9 | 22.7 | 23.8 | 22.7 | 22.9 |
| Range | 16.8–36.3 | 15.2–36.3 | 15.6–34.2 | 17.0–34.6 | 15.6–33.3 | 15.2–36.3 |
| P25, P75 | 20.6, 24.8 | 20.8, 24.9 | 20.8, 24.7 | 21.1, 25.5 | 20.6, 25.0 | 20.8, 24.9 |
| Marriage status (n, %) |  |  |  |  |  |  |
| Single | 4 (3.1) | 9 (3.8) | 7 (2.4) | 5 (4.1) | 7 (4.0) | 22 (3.5) |
| Married | 122 (93.1) | 224 (94.1) | 282 (94.9) | 116 (94.3) | 166 (94.9) | 584 (94.2) |
| Divorced | 2 (1.5) | 3 (1.3) | 3 (1.0) | 2 (1.6) | 0 (0) | 5 (0.8) |
| Widowed | 3 (2.3) | 2 (0.8) | 5 (1.7) | 0 (0) | 2 (1.1) | 9 (1.5) |
| Surgery category (n, %) |  |  |  |  |  |  |
| Conservation surgery | 60 (45.8) | 105 (44.1) | 112 (37.7) | 81 (65.8) | 68 (38.9) | 263 (42.4) |
| Mastectomy | 71 (54.2) | 133 (55.9) | 185 (62.3) | 42 (34.1) | 107 (61.1) | 357 (57.6) |
| Pathological stage (n, %) |  |  |  |  |  |  |
| Stage I | 56 (42.7) | 106 (44.5) | 103 (34.7) | 38 (30.9) | 50 (28.6) | 226 (36.5) |
| Stage II | 58 (44.3) | 103 (43.3) | 146 (49.2) | 62 (50.4) | 92 (52.6) | 300 (48.4) |
| Stage III | 17 (13.0) | 29 (12.2) | 48 (16.2) | 23 (18.7) | 33 (18.9) | 94 (15.2) |
| Histologic type (n, %) |  |  |  |  |  |  |
| Ductal | 119 (90.8) | 222 (93.3) | 279 (93.9) | 120 (97.6) | 166 (94.9) | 586 (94.5) |
| Lobular | 2 (1.5) | 2 (0.8) | 3 (1.0) | 1 (0.8) | 3 (1.7) | 7 (1.1) |
| Other | 10 (7.6) | 14 (5.9) | 15 (5.1) | 2 (1.6) | 6 (3.4) | 27 (4.4) |
| ECOG performance status (n, %) |  |  |  |  |  |  |
| Score = 0 | 122 (93.1) | 57 (23.9) | 107 (36.0) | 71 (57.7) | 95 (54.3) | 309 (49.8) |
| Score = 1 | 8 (6.1) | 179 (75.2) | 185 (62.3) | 52 (42.3) | 73 (41.7) | 298 (48.1) |
| Score = 2 | 1 (0.8) | 2 (0.8) | 5 (1.7) | 0 (0) | 7 (4.0) | 13 (2.1) |

Abbreviations: ECOG, Eastern Cooperative Oncology Group; *SD*, standard deviation.
