# Supplementary material for: Chinese Medicine Syndrome Differentiation for Early Breast Cancer: A Multicenter Prospective Clinical Study
Source: Front Oncol. 2022 Jul 7;12:914805. doi: 10.3389/fonc.2022.914805 (PMC9300931; doi:10.3389/fonc.2022.914805)
Supplement: Supplementary file 4 [file Table_3.docx]

Supplementary File 3: Syndromes in each breast cancer treatment stage.

Table S3.1. Syndromes in women in the preoperative stage.

| **Syndrome** | **Frequency (*n*)** | **Percentage (%)** |
| --- | --- | --- |
| Liver stagnation with congealing phlegm | 104 | 79.4 |
| Disharmony of *Chong* and *Ren* Vessels | 19 | 14.5 |
| Blood stasis with phlegm | 3 | 2.3 |
| Depressed Liver *qi* transforming into fire | 1 | 0.8 |
| Dual deficiency of the Liver and Kidney | 1 | 0.8 |
| Spleen and Stomach disharmony | 1 | 0.8 |
| Spleen deficiency with dampness and heat | 1 | 0.8 |
| Spleen deficiency with dampness encumbrance | 1 | 0.8 |

Table S3.2. Syndrome in women in the postoperative stage.

| **Syndrome** | **Frequency (*n*)** | **Percentage (%)** |
| --- | --- | --- |
| Spleen and Stomach disharmony | 141 | 59.2 |
| Dual deficiency of *qi* and Blood | 31 | 13.0 |
| Dual deficiency of *qi* and *yin* | 29 | 12.2 |
| Spleen and Stomach weakness | 27 | 11.3 |
| *Qi* stagnation and Blood stasis | 5 | 2.1 |
| Liver and Kidney *yin* deficiency | 1 | 0.4 |
| Liver depression and Blood stasis | 1 | 0.4 |
| Liver stagnation with congealing phlegm | 1 | 0.4 |
| Spleen and Stomach deficiency cold | 1 | 0.4 |
| Spleen deficiency with dampness encumbrance | 1 | 0.4 |

Table S3.3. Syndrome in women in the chemotherapy stage.

| **Syndrome** | **Frequency (*n*)** | **Percentage (%)** |
| --- | --- | --- |
| Dual deficiency of *qi* and Blood | 124 | 41.8 |
| Spleen and Stomach disharmony | 63 | 21.2 |
| Dual deficiency of *qi* and *yin* | 42 | 14.1 |
| Dual deficiency of the Spleen and Kidney | 23 | 7.7 |
| Dual deficiency of the Liver and Kidney | 15 | 5.1 |
| Liver depression and Spleen deficiency | 5 | 1.7 |
| Heart vessel obstruction | 4 | 1.3 |
| Spleen and Kidney *yang* deficiency | 2 | 0.7 |
| Spleen deficiency with dampness encumbrance | 2 | 0.7 |
| Spleen *qi* deficiency | 2 | 0.7 |
| Depressed Liver *qi* transforming into fire | 1 | 0.3 |
| Dual deficiency of the Heart and Kidney | 1 | 0.3 |
| Hyperactivity of Liver with Spleen deficiency | 1 | 0.3 |
| Kidney *yin* deficiency | 1 | 0.3 |
| Liver and Kidney *yin* deficiency | 1 | 0.3 |
| Liver depression and Blood stasis | 1 | 0.3 |
| Non-interaction between the Heart and Kidney | 1 | 0.3 |
| *Qi* deficiency with Blood stasis | 1 | 0.3 |
| *Qi* depression with congealing phlegm | 1 | 0.3 |
| *Qi* stagnation and Blood stasis | 1 | 0.3 |
| Spleen deficiency with dampness encumbrance, dual deficiency of *qi* and Blood | 1 | 0.3 |
| Spleen *yang* deficiency | 1 | 0.3 |
| Wind–cold fettering the exterior | 1 | 0.3 |
| *Yang* deficiency with water flood | 1 | 0.3 |
| *Yin* deficiency with *yang* floating | 1 | 0.3 |

Table S3.4. Syndrome in women in the radiation therapy stage.

| **Syndrome** | **Frequency (*n*)** | **Percentage (%)** |
| --- | --- | --- |
| Dual deficiency of *qi* and *yin* | 39 | 31.7 |
| Dual deficiency of *qi* and Blood | 35 | 28.5 |
| *Yin* deficiency with fluid depletion | 22 | 17.9 |
| *Yin* deficiency with fire toxin | 18 | 14.6 |
| Dampness and heat syndrome | 1 | 0.8 |
| Deficiency of healthy *qi* and exuberance of pathogen | 1 | 0.8 |
| Intense Heart fire | 1 | 0.8 |
| Lung and Stomach *yin* deficiency | 1 | 0.8 |
| Spleen and Stomach disharmony | 1 | 0.8 |
| Spleen and Stomach weakness | 1 | 0.8 |
| Spleen deficiency and Blood stasis | 1 | 0.8 |
| Spleen deficiency with dampness encumbrance | 1 | 0.8 |
| Spleen *qi* deficiency | 1 | 0.8 |

Table S3.5. Syndrome in women in the endocrine therapy stage.

| **Syndrome** | **Frequency (*n*)** | **Percentage (%)** |
| --- | --- | --- |
| Dual deficiency of *qi* and *yin* | 33 | 18.9 |
| Dual deficiency of the Spleen and Kidney | 30 | 17.1 |
| Dual deficiency of *qi* and Blood | 16 | 9.1 |
| Deficiency of healthy *qi* and exuberance of pathogen | 11 | 6.3 |
| Liver depression and Spleen deficiency | 11 | 6.3 |
| Spleen *qi* deficiency | 10 | 5.7 |
| Dual deficiency of the Liver and Kidney | 9 | 5.1 |
| Liver and Kidney *yin* deficiency | 9 | 5.1 |
| Disharmony of *Chong* and *Ren* Vessels | 5 | 2.9 |
| *Yin* deficiency with fire toxin | 5 | 2.9 |
| Deficiency of healthy *qi* and exuberance of toxin | 3 | 1.7 |
| Depressed Liver *qi* transforming into fire | 3 | 1.7 |
| Kidney *yin* deficiency | 3 | 1.7 |
| Effulgent Heart and Liver fire | 2 | 1.1 |
| Liver depression and *qi* stagnation | 2 | 1.1 |
| Non-interaction between the Heart and Kidney | 2 | 1.1 |
| *Qi* deficiency with Blood stasis | 2 | 1.1 |
| Spleen and Stomach disharmony | 2 | 1.1 |
| Spleen and Stomach weakness | 2 | 1.1 |
| Spleen deficiency with dampness encumbrance | 2 | 1.1 |
| Dampness and heat syndrome | 1 | 0.6 |
| Dual deficiency of the Heart and Spleen | 1 | 0.6 |
| Heart deficiency with timidity | 1 | 0.6 |
| Heart *yang* deficiency | 1 | 0.6 |
| Heart *yin* deficiency | 1 | 0.6 |
| Kidney deficiency | 1 | 0.6 |
| Kidney *qi* insecurity | 1 | 0.6 |
| Lesser *yang* (*Shao yang*) disharmony | 1 | 0.6 |
| Liver depression and Blood deficiency | 1 | 0.6 |
| Liver stagnation with congealing phlegm | 1 | 0.6 |
| Lung and Kidney *qi* deficiency | 1 | 0.6 |
| Phlegm–dampness with stagnated heat | 1 | 0.6 |
| Spleen and Kidney *yin* deficiency | 1 | 0.6 |
