# Supplementary material for: Chinese Medicine Syndrome Differentiation for Early Breast Cancer: A Multicenter Prospective Clinical Study
Source: Front Oncol. 2022 Jul 7;12:914805. doi: 10.3389/fonc.2022.914805 (PMC9300931; doi:10.3389/fonc.2022.914805)
Supplement: Supplementary file 5 [file Table_4.docx]

Supplementary File 4: Syndromes by pathological stage in each breast cancer treatment stage.

**Table S4.1. Syndromes by pathological stage in the preoperative stage (n = 131).**

| **Syndrome** | **Pathological stage I *n* (%)** | **Pathological stage II *n* (%)** | **Pathological stage III *n* (%)** |
| --- | --- | --- | --- |
| Liver stagnation with congealing phlegm | 48 (36.6) | 44 (33.6) | 12 (9.2) |
| Disharmony of *Chong* and *Ren* Vessels | 8 (6.1) | 8 (6.1) | 3 (2.3) |
| Blood stasis with phlegm | 0 (0) | 2 (1.5) | 1 (0.8) |
| Depressed Liver *qi* transforming into fire | 0 (0) | 1 (0.8) | 0 (0) |
| Dual deficiency of the Liver and Kidney | 0 (0) | 1 (0.8) | 0 (0) |
| Spleen and Stomach disharmony | 0 (0) | 1 (0.8) | 0 (0) |
| Spleen deficiency with dampness and heat | 0 (0) | 0 (0) | 1 (0.8) |
| Spleen deficiency with dampness encumbrance | 0 (0) | 1 (0.8) | 0 (0) |

**Table S4.2. Syndromes by pathological stage in the postoperative stage (n = 238).**

| **Syndrome** | **Pathological stage I *n* (%)** | **Pathological stage II *n* (%)** | **Pathological stage III *n* (%)** |
| --- | --- | --- | --- |
| Spleen and Stomach disharmony | 61 (25.6) | 62 (26.1) | 18 (7.6) |
| Dual deficiency of *qi* and Blood | 15 (6.3) | 15 (6.3) | 1 (0.4) |
| Dual deficiency of *qi* and *yin* | 16 (6.7) | 9 (3.8) | 4 (1.7) |
| Spleen and Stomach weakness | 9 (3.8) | 13 (5.5) | 5 (2.1) |
| *Qi* stagnation and Blood stasis | 3 (1.3) | 2 (0.8) | 0 (0) |
| Liver and Kidney *yin* deficiency | 1 (0.4) | 0 (0) | 0 (0) |
| Liver depression and Blood stasis | 0 (0) | 0 (0) | 1 (0.4) |
| Liver stagnation with congealing phlegm | 0 (0) | 1 (0.4) | 0 (0) |
| Spleen and Stomach deficiency cold | 0 (0) | 1 (0.4) | 0 (0) |
| Spleen deficiency with dampness encumbrance | 1 (0.4) | 0 (0) | 0 (0) |

**Table S4.3. Syndromes by pathological stage in the chemotherapy stage (n = 297).**

| **Syndrome** | **Pathological stage I *n* (%)** | **Pathological stage II *n* (%)** | **Pathological stage III *n* (%)** |
| --- | --- | --- | --- |
| Dual deficiency of *qi* and Blood | 40 (13.5) | 70 (23.6) | 14 (4.7) |
| Spleen and Stomach disharmony | 24 (8.1) | 29 (9.8) | 10 (3.4) |
| Dual deficiency of *qi* and *yin* | 13 (4.4) | 18 (6.1) | 11 (3.7) |
| Dual deficiency of the Spleen and Kidney | 8 (2.7) | 11 (3.7) | 4 (1.3) |
| Dual deficiency of the Liver and Kidney | 8 (2.7) | 2 (0.7) | 5 (1.7) |
| Liver depression and Spleen deficiency | 3 (1.0) | 2 (0.7) | 0 (0) |
| Heart vessel obstruction | 0 (0) | 3 (1.0) | 1 (0.3) |
| Spleen and Kidney *yang* deficiency | 0 (0) | 2 (0.7) | 0 (0) |
| Spleen deficiency with dampness encumbrance | 1 (0.3) | 1 (0.3) | 0 (0) |
| Spleen *qi* deficiency | 1 (0.3) | 0 (0) | 1 (0.3) |
| Depressed Liver *qi* transforming into fire | 0 (0) | 0 (0) | 1 (0.3) |
| Dual deficiency of the Heart and Kidney | 1 (0.3) | 0 (0) | 0 (0) |
| Hyperactivity of Liver with Spleen deficiency | 0 (0) | 1 (0.3) | 0 (0) |
| Kidney *yin* deficiency | 0 (0) | 0 (0) | 1 (0.3) |
| Liver and Kidney *yin* deficiency | 0 (0) | 1 (0.3) | 0 (0) |
| Liver depression and Blood stasis | 1 (0.3) | 0 (0) | 0 (0) |
| Non-interaction between the Heart and Kidney | 0 (0) | 1 (0.3) | 0 (0) |
| *Qi* deficiency with Blood stasis | 0 (0) | 1 (0.3) | 0 (0) |
| *Qi* depression with congealing phlegm | 1 (0.3) | 0 (0) | 0 (0) |
| *Qi* stagnation and Blood stasis | 1 (0.3) | 0 (0) | 0 (0) |
| Spleen deficiency with dampness encumbrance, dual deficiency of *qi* and Blood | 0 (0) | 1 (0.3) | 0 (0) |
| Spleen *yang* deficiency | 0 (0) | 1 (0.3) | 0 (0) |
| Wind–cold fettering the exterior | 1 (0.3) | 0 (0) | 0 (0) |
| *Yang* deficiency with water flood | 0 (0) | 1 (0.3) | 0 (0) |
| *Yin* deficiency with *yang* floating | 0 (0) | 1 (0.3) | 0 (0) |

**Table S4.4. Syndromes by pathological stage in the radiation therapy stage (n = 123).**

| **Syndrome** | **Pathological stage I *n* (%)** | **Pathological stage II *n* (%)** | **Pathological stage III *n* (%)** |
| --- | --- | --- | --- |
| Dual deficiency of *qi* and *yin* | 8 (6.5) | 21 (17.1) | 10 (8.1) |
| Dual deficiency of *qi* and Blood | 12 (9.8) | 18 (14.6) | 5 (4.1) |
| *Yin* deficiency with fluid depletion | 12 (9.8) | 9 (7.3) | 1 (0.8) |
| *Yin* deficiency with fire toxin | 4 (3.3) | 10 (8.1) | 4 (3.3) |
| Dampness and heat syndrome | 1 (0.8) | 0 (0) | 0 (0) |
| Deficiency of healthy *qi* and exuberance of pathogen | 0 (0) | 0 (0) | 1 (0.8) |
| Intense Heart fire | 0 (0) | 1 (0.8) | 0 (0) |
| Lung and Stomach *yin* deficiency | 0 (0) | 0 (0) | 1 (0.8) |
| Spleen and Stomach disharmony | 1 (0.8) | 0 (0) | 0 (0) |
| Spleen and Stomach weakness | 0 (0) | 0 (0) | 1 (0.8) |
| Spleen deficiency and Blood stasis | 0 (0) | 1 (0.8) | 0 (0) |
| Spleen deficiency with dampness encumbrance | 0 (0) | 1 (0.8) | 0 (0) |
| Spleen *qi* deficiency | 0 (0) | 1 (0.8) | 0 (0) |

**Table S4.5. Syndromes by pathological stage in the endocrine therapy stage (n = 175).**

| **Syndrome** | **Pathological stage I *n* (%)** | **Pathological stage II *n* (%)** | **Pathological stage III *n* (%)** |
| --- | --- | --- | --- |
| Dual deficiency of *qi* and *yin* | 11 (6.3) | 18 (10.3) | 4 (2.3) |
| Dual deficiency of the Spleen and Kidney | 9 (5.1) | 13 (7.4) | 8 (4.6) |
| Dual deficiency of *qi* and Blood | 7 (4.0) | 7 (4.0) | 2 (1.1) |
| Deficiency of health *qi* and exuberance of pathogen | 0 (0) | 10 (5.7) | 1 (0.6) |
| Liver depression and Spleen deficiency | 6 (3.4) | 5 (2.9) | 0 (0) |
| Spleen *qi* deficiency | 0 (0) | 7 (4.0) | 3 (1.7) |
| Dual deficiency of the Liver and Kidney | 3 (1.7) | 3 (1.7) | 3 (1.7) |
| Liver and Kidney *yin* deficiency | 3 (1.7) | 5 (2.9) | 1 (0.6) |
| Disharmony of *Chong* and *Ren* Vessels | 2 (1.1) | 1 (0.6) | 2 (1.1) |
| *Yin* deficiency with fire toxin | 1 (0.6) | 3 (1.7) | 1 (0.6) |
| Deficiency of health *qi* and exuberance of toxin | 0 (0) | 2 (1.1) | 1 (0.6) |
| Depressed Liver *qi* transforming into fire | 1 (0.6) | 1 (0.6) | 1 (0.6) |
| Kidney *yin* deficiency | 0 (0) | 3 (1.7) | 0 (0) |
| Effulgent Heart and Liver fire | 0 (0) | 1 (0.6) | 1 (0.6) |
| Liver depression and *qi* stagnation | 0 (0) | 2 (1.1) | 0 (0) |
| Non-interaction between the Heart and Kidney | 1 (0.6) | 0 (0) | 1 (0.6) |
| *Qi* deficiency with Blood stasis | 1 (0.6) | 0 (0) | 1 (0.6) |
| Spleen and Stomach disharmony | 0 (0) | 2 (1.1) | 0 (0) |
| Spleen and Stomach weakness | 0 (0) | 1 (0.6) | 1 (0.6) |
| Spleen deficiency with dampness encumbrance | 0 (0) | 2 (1.1) | 0 (0) |
| Dampness and heat syndrome | 1 (0.6) | 0 (0) | 0 (0) |
| Dual deficiency of the Heart and Spleen | 0 (0) | 1 (0.6) | 0 (0) |
| Heart deficiency with timidity | 0 (0) | 1 (0.6) | 0 (0) |
| Heart *yang* deficiency | 0 (0) | 0 (0) | 1 (0.6) |
| Heart *yin* deficiency | 1 (0.6) | 0 (0) | 0 (0) |
| Kidney deficiency | 1 (0.6) | 0 (0) | 0 (0) |
| Kidney *qi* insecurity | 1 (0.6) | 0 (0) | 0 (0) |
| Lesser *yang* (*Shao yang*) disharmony | 1 (0.6) | 0 (0) | 0 (0) |
| Liver depression and Blood deficiency | 0 (0) | 1 (0.6) | 0 (0) |
| Liver stagnation with congealing phlegm | 0 (0) | 1 (0.6) | 0 (0) |
| Lung and Kidney *qi* deficiency | 0 (0) | 1 (0.6) | 0 (0) |
| Phlegm–dampness with stagnated heat | 0 (0) | 0 (0) | 1 (0.6) |
| Spleen and Kidney *yin* deficiency | 0 (0) | 1 (0.6) | 0 (0) |
