# Supplementary material for: Chinese Medicine Syndrome Differentiation for Early Breast Cancer: A Multicenter Prospective Clinical Study
Source: Front Oncol. 2022 Jul 7;12:914805. doi: 10.3389/fonc.2022.914805 (PMC9300931; doi:10.3389/fonc.2022.914805)
Supplement: Supplementary file 6 [file Table_5.docx]

Supplementary File 5: Chinese herbal medicine (CHM) formulas in each breast cancer treatment stage.

Table S5.1. Frequency of CHM formulas for the preoperative stage.

| **Syndrome** | **Formula** | **Frequency** |
| --- | --- | --- |
| Depressed Liver *qi* transforming into fire | *Dan zhi xiao yao san* | 1 |
| Liver stagnation with congealing phlegm | *Xiao yao lou bei san* | 63 |
|  | *Chai hu shu gan san* | 1 |
| Spleen and Stomach disharmony | *Xiang sha liu jun zi tang* | 1 |

**Table S5.2. Frequency of CHM formulas for the postoperative stage.**

| **Syndrome** | **Formula** | **Frequency** |
| --- | --- | --- |
| Dual deficiency of *qi* and Blood | *Shen ling bai zhu san* | 6 |
|  | *Gui pi tang* | 2 |
|  | *Ba zhen tang* | 1 |
|  | *Clinician-developed formula** | 1 |
|  | *Si jun zi tang* | 1 |
| Dual deficiency of *qi* and *yin* | *Sheng mai san* | 1 |
| Liver depression and Blood stasis | *Clinician-developed formula** | 1 |
| *Qi* stagnation and Blood stasis | *Clinician-developed formula** | 2 |
|  | *Xue fu zhu yu tang* | 2 |
|  | *Ba zhen tang* | 1 |
| Spleen and Stomach disharmony | *Xiang sha liu jun zi tang* | 126 |
|  | *Shen ling bai zhu san* | 1 |
| Spleen and Stomach weakness | *Xiang sha liu jun zi tang* | 27 |
| Spleen and Stomach deficiency cold | *Wen jing tang* and *Xiao chai hu tang* | 1 |
| Spleen deficiency with dampness encumbrance | *Shen ling bai zhu san* | 1 |

Note: “Clinician-developed formula” refers to formulas developed by the practitioner based on personal experience.

**Table S5.3.** **Frequency of CHM formulas for the chemotherapy stage.**

| **Syndrome** | **Formula** | **Frequency** |
| --- | --- | --- |
| Dual deficiency of *qi* and Blood | *Gui pi tang* | 23 |
|  | *Clinician-developed formula** | 22 |
|  | *Si jun zi tang* | 11 |
|  | *Gui pi tang* and *Gui lu er xian tang* | 8 |
|  | *Ba zhen tang* | 5 |
|  | *Shen ling bai zhu san* | 4 |
|  | *Shen xiao gua lou san* and *Kai yu san* | 2 |
| Dual deficiency of *qi* and *yin* | *Clinician-developed formula** | 1 |
| Wind–cold fettering the exterior | *Xiao qing long tang* and *Si shen wan* | 1 |
| Heart and Kidney deficiency | *Gan mai da zao tang* and *Suo quan wan* | 1 |
| Heart–Kidney non-interaction | *Jiao tai wan* and *Suo quan wan* | 1 |
| Heart vessel obstruction | *Clinician-developed formula** | 3 |
|  | *Tao ren hong hua jian* | 1 |
| Hyperactivity of Liver with Spleen deficiency | *Clinician-developed formula** | 1 |
| Kidney *yin* deficiency | *Clinician-developed formula** | 1 |
| Liver and Kidney *yin* deficiency | *Zhi bai di huang wan* | 1 |
| Liver depression and Blood stasis | *Clinician-developed formula** | 1 |
| *Qi* deficiency with Blood stasis | *Xue fu zhu yu tang* | 1 |
| *Qi* stagnation and Blood stasis | *Clinician-developed formula** | 1 |
| *Qi* stagnation with congealing phlegm | *Er chen tang* | 1 |
| Spleen and Kidney *yang* deficiency | *Clinician-developed formula** | 1 |
| Spleen and Stomach disharmony | *Xiang sha liu jun zi tang* | 4 |
|  | *Clinician-developed formula** | 1 |
|  | *Li zhong tang* and *Wu zhu yu tang* | 1 |
|  | *Yi gong san* | 1 |
| Spleen deficiency with dampness encumbrance | *Wu ling san* | 1 |
| Spleen deficiency with dampness encumbrance, dual deficiency of *qi* and Blood | *Clinician-developed formula** | 1 |
| Spleen *qi* deficiency | *Clinician-developed formula** | 1 |
|  | *Yi gong san* and *Liu wei di huang wan* | 1 |
| *Yin* deficiency with *yang* floating | *Clinician-developed formula** | 1 |

Note: “Clinician-developed formula” refers to formulas developed by the practitioner based on personal experience.

**Table S5.4. Frequency of CHM formulas for the radiation therapy stage.**

| **Syndrome** | **Formula** | **Frequency** |
| --- | --- | --- |
| Dampness and heat syndrome | *Yin chen hao tang* | 1 |
| Deficiency of healthy *qi* and exuberance of pathogen | *Huang qi jie du tang* | 1 |
| Dual deficiency of *qi* and Blood | *Gui pi tang* | 28 |
|  | *Si jun zi tang* | 1 |
| Dual deficiency of *qi* and *yin* | *Sheng mai san* | 10 |
|  | *Sha shen mai dong tang* | 1 |
|  | *Shen ling bai zhu san* | 1 |
| Intense Heart fire | *Clinician-developed formula** | 1 |
| Lung and Stomach *yin* deficiency | *Sha shen mai dong tang* | 1 |
| Spleen and Stomach disharmony | *Chen xia liu jun zi tang* | 1 |
| Spleen and Stomach weakness | *Xiang sha liu jun zi tang* | 1 |
| Spleen *qi* deficiency | *Yi gong san* | 1 |
| *Yin* deficiency with fluid depletion | *Sha shen mai dong tang* | 1 |

Note: “Clinician-developed formula” refers to formulas developed by the practitioner based on personal experience.

**Table S5.5.** **Frequency of CHM formulas for the endocrine therapy stage.**

| **Syndrome** | **Formula** | **Frequency** |
| --- | --- | --- |
| Dampness and heat syndrome | *Clinician-developed formula** | 1 |
| Deficiency of healthy *qi* and exuberance of pathogen | *Huang qi jie du tang* | 10 |
|  | *Jing fang bai du san* | 1 |
| Deficiency of healthy *qi* and exuberance of toxin | *Ba zhen tang* | 1 |
|  | *Si jun zi tang* | 1 |
| Depressed Liver *qi* transforming into fire | *Chai hu shu gan san* | 1 |
| Dual deficiency of *qi* and Blood | *Clinician-developed formula** | 4 |
|  | *Gui pi tang* | 2 |
|  | *Ba zhen tang* | 1 |
|  | *Shen ling bai zhu san* | 1 |
| Dual deficiency of *qi* and *yin* | *Sheng mai san* | 1 |
|  | *Sheng mai san* and *Jiao tai wan* | 1 |
|  | *Si jun zi tang* and *Gan mai da zao tang* | 1 |
| Effulgent Heart–Liver fire | *Clinician-developed formula** | 2 |
| Heart and Spleen deficiency | *Yi gong san* | 1 |
| Heart deficiency with timidity | *Yu ping feng san* and *Gan mai da zao tang* | 1 |
| Heart–Kidney non-interaction | *Clinician-developed formula** | 1 |
|  | *Gan mai da zao tang* | 1 |
| Heart *yang* deficiency | *Gui zhi gan cao long gu mu li tang* | 1 |
| Insecurity of Kidney *qi* | *Suo quan wan* | 1 |
| Lesser *yang* disharmony | *Xiao chai hu tang* | 1 |
| Liver and Kidney deficiency | *Liu wei di huang wan* | 1 |
| Liver and Kidney *yin* deficiency | *Clinician-developed formula** | 2 |
|  | *Liu wei di huang wan* | 1 |
| Liver depression and Spleen deficiency | *Si jun zi tang* | 1 |
| *Qi* deficiency with Blood stasis | *Clinician-developed formula** | 1 |
|  | *Yi gong san* | 1 |
| Spleen and Kidney deficiency | *Si jun zi tang* | 1 |
|  | *Si jun zi tang* and *Er xian tang* | 1 |
|  | *Yi gong san* | 1 |
|  | *Yi gong san* and *Suo quan wan* | 1 |
|  | *Yu ping feng san* and *Suo quan wan* | 1 |
| Spleen and Stomach weakness | *Yi gong san* | 2 |
| Spleen deficiency with dampness encumbrance | *Shen ling bai zhu san* | 1 |
|  | *Wu ling san* | 1 |
| Spleen *qi* deficiency | *Si jun zi tang* | 2 |
|  | *Yi gong san* | 2 |
|  | *Clinician-developed formula** | 1 |
|  | *Yi gong san* and *Gan mai da zao tang* | 1 |
|  | *Yi gong san* and *Liu wei di huang wan* | 1 |
| *Yin* deficiency with fire toxin | *Gan mai da zao tang* | 3 |
|  | *Clinician-developed formula** | 1 |

Note: “Clinician-developed formula” refers to formulas developed by the practitioner based on personal experience.
